# Effects of an Omega-3 Supplemented, High-Protein Diet in Combination with Vibration and Resistance Exercise on Muscle Power and Inflammation in Old Adults: A Pilot Randomized Controlled Trial

**DOI:** 10.3390/nu14204274

**Published:** 2022-10-13

**Authors:** Ulrike Haß, Bastian Kochlik, Catrin Herpich, Stefan Rudloff, Kristina Norman

**Affiliations:** 1 Department of Nutrition and Gerontology, German Institute of Human Nutrition Potsdam-Rehbruecke, 14558 Nuthetal, Germany; 2 Institute of Nutritional Science, University of Potsdam, 14558 Nuthetal, Germany; 3 Department of Geriatrics and Medical Gerontology, Charité—Universitätsmedizin Berlin, Corporate Member of Freie Universität Berlin and Humboldt-Universität zu Berlin, 13347 Berlin, Germany; 4 Institute of Sports Science, Martin Luther University of Halle-Wittenberg, 06108 Halle (Saale), Germany; 5 German Center for Cardiovascular Research (DZHK), Partner Site Berlin, 10115 Berlin, Germany

**Keywords:** inflammaging, healthy aging, omega-3, high protein, muscle power, chair rise test, mechanography, whole body vibration

## Abstract

Background: Inflammaging is considered to drive loss of muscle function. Omega-3 fatty acids exhibit anti-inflammatory properties. Therefore, we examined the effects of eight weeks of vibration and home-based resistance exercise combined with a whey-enriched, omega-3-supplemented diet on muscle power, inflammation and muscle biomarkers in community-dwelling old adults. Methods: Participants were randomized to either exercise (3x/week, *n* = 20), exercise + high-protein diet (1.2–1.5 g/kg, *n* = 20), or exercise + high-protein and omega-3-enriched diet (2.2 g/day, *n* = 21). Muscle power (watt/m^2^) and chair rise test (CRT) time (s) were assessed via CRT measured with mechanography. Furthermore, leg strength (kg/m^2^) and fasting concentrations of inflammatory (interleukin (IL-) 6, IL-10, high-mobility group box-1 (HMGB-1)) and muscle biomarkers (insulin-like growth factor (IGF-) 1, IGF-binding protein-3, myostatin) were assessed. Results: Sixty-one participants (70.6 ± 4.7 years; 47% men) completed the study. According to generalized linear mixed models, a high-protein diet improved leg strength and CRT time. Only IGF-1 increased with additional omega-3. Sex-specific analyses revealed that muscle power, IL-6, IL-6/IL-10 ratio, and HMGB-1 improved significantly in the male high-protein, omega-3-enriched group only. Conclusion: Vibration and home-based resistance exercise combined with a high-protein, omega-3-enriched diet increased muscle power and reduced inflammation in old men, but not in old women. While muscle biomarkers remained unchanged, a high-protein diet combined with exercise improved leg strength and CRT time.

## 1. Introduction

Aging is associated with a decline in muscle function. One contributing factor to this multi-factorial process is inflammaging [1]. Chronic inflammatory processes are known to activate proteolytic pathways and inhibit growth factors [2], and are predictive of lower muscle strength and muscle power in old adults [3]. Muscle power is determined by neuromuscular function [4] and exhibits an earlier and a more pronounced decline with age compared to muscle mass and strength [5]. Since movement is the action of force along a distance in a certain time, muscle power (= force × velocity) rather than mere muscle strength is the key element of functionality and determines fall risk [6] and mobility limitations [7]. In particular, sit-to-stand transitions are described as “the most important neuromuscular risk factor for falls and fall-related fractures” [8] (p. 167). Therefore, chair rise tests (CRT) have prognostic value regarding individual fall risk in community-dwelling old adults [9] and are related to limitations in activities of daily living [10], which in turn are associated with lower quality of life and higher health care costs [10]. While resistance exercise is essential to preserve muscle strength and function, vibration exercise improves muscle power [11].

Aged muscles show a blunted response on protein intake, which is known as anabolic resistance [12]. Accordingly, ≥1.2 g protein/kg body weight (bw)/day is recommended in older-aged individuals; and in particular, whey protein is known as potent anabolic stimulus due to its high leucine content [13]. Furthermore, a diet-induced higher inflammatory burden is associated with systemic inflammation [14] as well as with reduced muscle mass and function in healthy old adults [15]. Omega-3 fatty acids have anti-inflammatory properties and have been shown to improve leg strength and CRT time in old adults [16]. Smith et al. [17] experimentally demonstrated that omega-3 supplementation (3.4 g/day) potentiates muscle protein synthesis in old adults following a hyper-aminoacidemic, hyper-insulinemic clamp. Different trials showed positive effects of omega-3 alone or combined with resistance exercise on lean mass, strength, or muscle function [16]. However, previous results from exercise and nutrition research are conflicting, and intervention studies combining vibration exercise and dietary modifications with omega-3 and protein supplementation that focus on muscle power in old adults still have to be performed [18]. Therefore, the primary aim of this pilot study was to estimate the effects of a high-protein diet supplemented with high-dose omega-3 fatty acids in combination with vibration and home-based resistance exercise on muscle power in old adults. In a second step, we evaluated how concomitant changes in inflammation were related to changes in muscle parameters. Further endpoints include leg strength, muscle biomarkers, and body composition.

## 2. Materials and Methods

### 2.1. Study Design and Population Sample

This 8-week open-labelled, randomized-controlled pilot trial was conducted in a 3-arm design with community-dwelling old adults (65–85 years) who were recruited via the institute’s website, flyers, newsletters, and newspaper ads (01/2020–01/2022). Neuromuscular adaptation and gains in muscle power occur within 2–8 weeks of resistance exercise [19], and likely occur with more efficacy in combination with vibration exercise [20]. Due to the anticipated synergistic effects of vibration exercise with nutritional intervention, and in order to ensure compliance, the intervention was conducted over eight weeks. The study was approved by the University of Potsdam ethics committee (protocol code 58/2019), registered at the German study register (DRKS00018995) and carried out in accordance with the Declaration of Helsinki. Exclusion criteria were smoking, any malignant or severe disease, diabetes mellitus type 1 and 2, dementia, or severe food intolerances regarding milk protein, lactose, seafood or algae. All participants gave written informed consent. Following an overnight fast, all measurements were performed before and after treatment at the German Institute of Human Nutrition Potsdam-Rehbruecke. Participants were further instructed to refrain from alcohol and vigorous exercise the day prior to examinations.

### 2.2. Dietary Intervention

Participants were randomly assigned to either control, high-protein, or high-protein plus omega-3 fatty acids. Simple randomization with random numbers were used to generate random allocation sequence. While the control group continued their usual diet, both protein-enriched groups received a daily 300 mL whey drink (27 g protein; including 4 g leucine; 136 kcal). They were instructed to evenly distribute their daily protein intake with 25–30 g protein/meal, with a goal of 1.2–1.5 g protein/kg bw/day, as this has been recommended by leading experts [13] to overcome anabolic resistance. To support a sufficient protein intake per meal, we assisted the participants with a scoring system that translated protein amounts of common foods to points (5 g protein = 1 point). Participants were further instructed to consume the whey drink within one hour after exercise on training days. Additionally, the high-protein, omega-3-enriched group was supplemented daily with 3.5 mL algae oil (2195 mg omega-3 fatty acids; including 1397 mg docosahexaenoic acid (DHA), 749 mg eicosapentaenoic acid (EPA), and 49 mg docosapentaenoic acid; 20 kcal) and instructed to take the oil with a fat-containing meal. The use of at least 2 g omega-3 fatty acids per day is deemed necessary to observe an effect in human interventional trials [18,21]. Compliance was evaluated by weighing left-overs of the supplements at the end of the study, as well as by measuring the omega-3 index in plasma.

### 2.3. Vibration and Home-Based Resistance Exercise

All participants received weekly vibration exercise on a Galileo^®^ side-alternating vibration plate (Novotec Medical GmbH, Pforzheim, Germany) under guidance at the institute. This included a one-minute warm-up phase with a frequency at 12 Hz, three minutes of dynamic and static squatting at an amplitude of 1.5–2 mm, and a one-minute cool-down phase at 12 Hz. Additionally, all participants received instructions for home-based resistance exercises, which had to be performed three times per week. These sessions of approximately 45 min included body weight training with three sets of seated crunches, marching, squats, chair rises, and chair dips, which were easily and safely implementable in everyday life with no further equipment needed aside from one chair. To account for individual physical conditions and accordingly avoid under- or overtraining during the intervention period, vibration frequency and number of repetitions of home-based exercises were evaluated individually for each participant at baseline. The training protocol for each participant thus started at their individual upper performance limit, followed by a weekly increase in vibration frequency (+2 Hz) and number of repetitions (+2) to ensure progression. Adherence to training protocols were documented with training diaries.

### 2.4. Anthropometric Measurements

Weight (kg), height (cm), and waist circumference (cm) were measured according to standard criteria to subsequently calculate body mass index (BMI) (kg/m^2^) and waist/height ratio. Body composition expressed as fat-free mass index (FFMI) (kg/m^2^) was estimated with single frequency bioimpedance analysis (Bioimpedance Analyzer Quantum/S Akern, Florence, Italy; with resolution on impedance components: resistance (Rz) 1% and reactance (Xc) 2% according to the manufacturer) at 50 kHz with the participants lying in the supine position and electrodes placed on the right hand and foot.

### 2.5. Muscle Power

Our primary outcome of interest, muscle power, was assessed digitally via CRT, using LEONARDO^®^ mechanography with a bench (height 46 cm) anchored to the force plate (Mechanograph^®^ GRFP STD, Novotec Medical GmbH, Pforzheim, Germany). During the CRT, participants moved five times as fast as possible with arms crossed from a seated to a standing position. CRT time (s) and muscle power normalized for height squared (watt/m^2^) were recorded.

### 2.6. Muscle Strength and Function

Knee extension strength (kg/m^2^) was measured in both legs by performing five seconds of maximal isometric contraction, while seated in a dynamometer chair (Iso-CheckMobil, DigiMax Systems, Hamm, Germany) with hips and knees at 90 degrees and arms crossed. Grip strength (kg/BMI) was measured according to a standardized approach [22] using LEONARDO^®^ mechanography (Mechanograph^®^ GF, Novotec Medical GmbH, Pforzheim, Germany). Four-meter gait speed (m/s) was measured at participants’ usual pace from a standing start.

### 2.7. Laboratory Assessments

Fasting blood samples were taken between 8–10 a.m. and stored at −80 °C until analysis. Serum C-reactive protein (CRP) (mg/L) was analysed using a colorimetric method (ABX Pentra 400, Horiba Ltd., Kyoto, Japan), while interleukin (IL-) 6 (pg/mL), IL-10 (pg/mL), high-mobility group box (HMGB)-1 (ng/mL), myostatin (ng/mL), insulin-like growth factor (IGF)-1 (ng/mL) and IGF-binding protein (IGFBP)-3 (mg/mL) concentrations were measured by using commercial immunosorbent assays (IL-6 inter-assay coefficients of variability (CV): 4.7–5.0%, intra-assay CV: 4.2–5.1%; IL-10 inter-assay CV: 3.7–4.8%, intra-assay CV: 1.9–2.0%; IGF-1 inter-assay CV: 5.53–6.56%, intra-assay CV: 5.08–6.65%; IGFBP-3 inter-assay CV: 3.0–6.8%, intra-assay CV: 2.5–5.6% (all BioVendor, Brno, Czech Republic); HMGB-1 inter-assay CV: 1.3–10.7%, intra-assay CV: 3.2–10.2% (IBL International GmbH, Hamburg, Germany); myostatin inter-assay CV: 3.1–6.0%, intra-assay CV: 1.8–5.4% (R&D Systems, Inc., Minneapolis, MN, USA)). IL-6 to IL-10 and IGF-1 to IGFBP-3 were also expressed as ratios, reflecting cytokine balance and IGF-1 bioavailability, respectively. Fatty acid spectrum was measured in plasma phospholipid fraction by gas chromatography as previously reported [23]. The omega-3 index represents the sum of EPA and DHA as percentage [%] of the total fatty acid spectrum.

### 2.8. Physical Activity

ActiGraph wGT3X-BT accelerometers (Actigraph Corp., Pensacola, FL, USA), worn on the right hip, were used to determine time spent in vigorous, moderate, low or sedentary activity (min./day) during the first and last study weeks. Participants were asked to record when the accelerometer was detached, e.g., when taking a shower or during swimming, to control for non-wear times. Data from at least four out of seven days (wear time ≥ six h/day) [24] were analysed with ActiLife 6.13.4 software (Actigraph Corp., Pensacola, FL, USA), using Freedson cut-points [25].

### 2.9. Dietary Assessment

Dietary intake was recorded using 3-day dietary protocols and calculated with the nutrition software EBISpro version 2016 (Dr. J. Erhart, Willstätt-Legelshurst, Germany).

### 2.10. Data Analysis

Statistical analyses were performed with SPSS Statistics version 25 (IBM Corp., Chicago, IL, USA). Data distribution was checked using Kolmogorov-Smirnov tests and Q-Q plots and presented as either mean ± standard deviation or median with interquartile range. Analysis of variance or Kruskal-Wallis tests were performed to compare continuous variables between the three groups at baseline. Differences in categorical variables were determined with Chi-square test. Within-group comparisons before and after treatment were tested with either paired *t*-test or Wilcoxon rank test. Associations between changes in dietary intakes, biomarker concentrations, muscle function, and body composition were determined with either Pearson (r) or Spearman (rho) correlations. 

Generalized linear mixed models with random effect on subjects to control for individual variance were used to investigate group-time interactions. Group × time-interaction effects were evaluated in comparison to the reference group (control) at baseline as well as between the high-protein and the high-protein, omega-3-enriched group, and can be interpreted as the difference in the groups’ regression line before and after treatment. All models were adjusted for age, sex, and physical activity, since we observed significant associations with muscle parameters and biomarkers. Due to skewness, biomarker concentrations have been log-transformed before analyses. Results are presented as beta regression coefficient (β) with robust standard error (SE). Statistical significance was assumed at *p* < 0.05.

## 3. Results

### 3.1. Baseline Characteristics

A total of 61 old adults were included in the final analysis (see flow chart Supplementary Materials Figure S1). Participants were 70.6 ± 4.7 years old (53% women) and baseline characteristics such as age, sex distribution, body composition, muscle parameters, and biomarker concentrations were similar between the three groups (Table 1). Dietary intakes, fitness conditions, and physical activity were also comparable between the groups (Supplementary Materials Table S1). 

### 3.2. Exercise Adherence, Dietary Compliance and Nutrient Intake

Participants of all three groups started with comparable vibration frequencies as well as exercise repetition numbers, and training protocols increased similarly between groups (Supplementary Materials Table S1).

Regarding whey supplementation, compliance rates were 98% and 96% in the high-protein group and the high-protein, omega-3-enriched group, respectively. Consequently, protein intakes increased significantly in both the high-protein (+0.76 ± 0.33 g/kg bw, *p* < 0.001) and the high-protein, omega-3-enriched group (+0.62 ± 0.35 g/kg bw, *p* < 0.001), resulting in 1.73 ± 0.27 g/kg/bw and 1.56 ± 0.36 g/kg/bw in the high-protein and the high-protein, omega-3-enriched group, respectively (Supplementary Materials Table S1). 

Regarding omega-3 supplementation, the compliance was 85% and the omega-3 plasma index increased by 3.7 ± 1.7% (*p* < 0.001) in the high-protein, omega-3-enriched group. At the same time, the omega-3 plasma index decreased in the control (−0.6 ± 1.0%; *p* = 0.015) and the high-protein group (−1.0 ± 1.4%; *p* = 0.006) (Table 1).

### 3.3. Higher Protein Intake Improved Leg Strength, CRT Time, and Fat-Free Mass

Changes in leg strength (r = 0.357; *p* = 0.005 and r = 0.370; *p* = 0.004), CRT time (r = −0.356; *p* = 0.005 and r = −0.359; *p* = 0.005), and FFMI (rho = 0.324, *p* = 0.011 and rho = 0.320, *p* = 0.012) correlated moderately with changes in protein and leucine intake.

Table 2 displays changes in muscle parameters and body composition that were associated with each intervention in adjusted mixed models. Both protein-enriched interventions showed significantly higher FFMI after eight weeks. Although leg strength increased in both the high-protein (+3.3 ± 4.8 kg/m^2^, *p* = 0.004) and the high-protein, omega-3-enriched group (+2.3 ± 4.4 kg/m^2^, *p* = 0.029) in unadjusted within-group comparisons (Table 1), a significant change compared to control was only observed for the high-protein group (Table 2). However, the high-protein group did not significantly change compared to the high-protein, omega-3-enriched group (Table 2). CRT time was significantly decreased in the high-protein compared to the control group, but not significantly different compared to the high-protein, omega-3-enriched group (Table 2).

### 3.4. Effects of Omega-3 Supplementation on Muscle Power, Inflammation, and IGF-1

Changes in muscle power correlated positively with increase in EPA intake (r = 0.269; *p* = 0.038) and almost significantly with increasing DHA intake (rho = 0.245; *p* = 0.059), but not with the increase in omega-3 plasma index (rho = 0.135; *p* = 0.302). Moreover, increase in muscle power correlated inversely with decrease in IL-6 concentrations (rho = −0.257; *p* = 0.047).

According to unadjusted within-group comparisons, muscle power increased significantly only in the high-protein, omega-3-enriched group (+20.0 ± 24.2 watt/m^2^, *p* = 0.001) (Table 1). However, no significant group-time interaction effects were observed in adjusted mixed model analyses (Table 2). 

Regarding inflammation, only the high-protein, omega-3-enriched group showed significantly decreased concentrations of IL-6, IL-10, and HMGB-1 in within-group comparisons (Table 1). Table 2 also displays adjusted changes in inflammation and muscle biomarkers according to each intervention. There was a trend, although non-significant, towards decreased IL-6 and IL-10 concentrations in the high-protein, omega-3-enriched group compared to control.

Unadjusted within-group comparisons showed that IGF-1 significantly increased only in the high-protein, omega-3-enriched group (Table 1). Moreover, in adjusted mixed model analyses, IGF-1 concentrations significantly increased with additional omega-3 supplementation compared to control (Table 2). However, neither IGFBP-3, IGF-1/IGFBP-3, nor myostatin concentrations showed significant changes in any of the models (Table 2).

### 3.5. Sex-Specific Differences in Muscle Parameters and Inflammation

Due to sex-specific associations with muscle parameters and inflammatory cytokine concentrations emerging in our previous models, we also performed the analyses separately for women and men. Supplementary Materials Tables S2 and S3 present the baseline values as well as effects of each intervention, separately displayed for female and male participants, respectively. Significant group-time interactions regarding muscle parameters occurred only in men (Supplementary Materials Table S3). Compared to control, a significant increase in muscle power as well as significant reductions in IL-6, IL-6/IL-10 ratio and HMGB-1 concentrations were observed in the male high-protein, omega-3-enriched group (Supplementary Materials Table S3). Comparing analyses between the high-protein and high-protein, omega-3-enriched groups confirmed higher muscle power, lower IL-6 and tendentially lower HMGB-1 after eight weeks with additional omega-3 supplementation in men (Supplementary Materials Table S3).

## 4. Discussion

This pilot trial in community-dwelling old adults resulted in increased leg strength and reduced CRT time after eight weeks of vibration and home-based resistance exercise, combined with a high-protein diet. Moreover, additional omega-3 supplementation improved muscle power and reduced IL-6 and IL-6/IL-10 ratio as well as HMGB-1 in male participants only. 

To the best of our knowledge, this is the first study investigating the combination of a high-protein and omega-3-enriched diet with vibration exercise in a three-arm design in community-dwelling old adults focusing on muscle power. Previously, two trials examined the effects of a 12-week resistance training (three times/week) model combined with a daily multi-nutrient supplement (whey, casein, creatine, vitamin D, and omega-3) on muscle mass, strength, and function in 32 sedentary [26] and 49 healthy old men [27]. Although effects could not be attributed to single nutrients, both trials observed significant intervention effects for lean mass, strength, and, regarding sedentary men, also for CRT time (−9%). Protein and omega-3 seem to act synergistically on muscle protein synthesis by augmenting amino acid transport [28], increasing protein kinase activity [17,28], and improving mitochondrial function [29].

### 4.1. Different Effects on Muscle Strength and Power

Regarding leg strength and CRT time, the high-protein group showed significant improvements compared to control in the whole sample as well as separately analysed in male participants. This significant effect was lost when compared to the omega-3 supplemented group (Table 2). Importantly, the male high-protein group showed worse CRT time and leg strength at baseline, which may explain a stronger increase in the high-protein group after eight weeks, at least compared to control (Supplementary Materials Table S3). However, additional omega-3 supplementation was not superior to protein supplementation alone in our study. Since plateau effects of protein anabolism are known [12], one explanation may be that maximal rates of muscle protein synthesis were already reached with protein intakes above 1.5 g/kg bw in both our protein-enriched groups. Furthermore, a recent meta-analysis concluded that omega-3 supplementation improves leg strength, but to a lesser extent when combined with resistance exercise, since exercise is already a strong anabolic stimulus [16].

Mixed models showed no significant improvement of muscle power in the whole study population, except for the male high-protein, omega-3-enriched group in sex-specific analyses. These intervention effects were further confirmed in direct comparison analyses between the high-protein and high-protein, omega-3-enriched group (Supplementary Materials Table S3). Moreover, changes in muscle power correlated positively with increased EPA as well as DHA intake, and inversely with decreased IL-6 concentrations. Importantly, although changes in omega-3 plasma index did not correlate with changes in IL-6 concentrations, a decline in IL-6 was exclusively observed in the high-protein, omega-3-enriched group. However, the beneficial effects of omega-3 fatty acids on muscle power are likely not only limited to their anti-inflammatory properties, as EPA and DHA exert different functions in skeletal muscle. While EPA is involved in muscle protein turnover, DHA is more relevant in neuromuscular functions [28]. Since muscle power is determined by neuromuscular function [4], the incorporation of omega-3 fatty acids in muscle and neuron membranes has been assumed to support the neuronal activation [28].

### 4.2. Impact on Growth Factors

IGF-1 increased significantly in our high-protein, omega-3-enriched group, which is in line with another observation after eight weeks of omega-3 supplementation in 62 middle-aged men with cardiovascular disease [30]. Although underlying mechanisms are largely unknown, one possible explanation attributes this to the anti-inflammatory properties of omega-3 fatty acids, since cytokines suppress anabolic signalling pathways [31]. 

Other muscle biomarkers remained unchanged in this study. Myostatin, for instance, is known as an inhibitor of skeletal muscle development, and suppression of myostatin has been shown to promote muscle growth [32]. However, since increased as well as decreased serum concentrations have been observed with aging, its contribution to the age-related loss of muscle mass and function is still under debate [32,33] and might therefore be of less relevance to muscle mass and function in this study.

### 4.3. No Significant Changes in the Control Group

Surprisingly, we observed no significant changes in muscle parameters, biomarkers or body composition in our control group. This is unexpected, since the control group received vibration and home-based resistance exercises at similar frequencies and amounts as the high-protein and high-protein, omega-3-enriched groups (Supplementary Materials Table S1). Home-based exercise programmes are beneficial in counteracting sedentariness and improving physical function, particularly in times with restricted activity, as has been shown during the COVID-19 pandemic [34]. However, compared to progressive, supervised strength training, home-based resistance exercise is a relatively mild-to-moderate intervention. In this regard, our exercise protocol may have lacked the intensity needed to gain significant improvements without any dietary intervention in well-functioning, community-dwelling old adults without physical limitations.

### 4.4. Sex-Specific Differences

Although our study is likely underpowered to detect sex-specific intervention effects, significant effects on muscle parameters and inflammation were only observed in men. Underlying mechanisms for sex-specific differences are complex and still under investigation, but are presumably attributed to sex-related genetic differences, concomitant hormonal milieu [35], and immune response [36], which also persist at older age. Furthermore, men exhibit a higher amount of type 2 muscle fibres and motor units [35], which result in greater strength and increased neuromuscular activation, i.e., muscle power. In our study, men showed greater muscle strength and power compared to women (Supplementary Materials Table S4). Moreover, visceral fat triggers a pro-inflammatory milieu [36]. In comparison to our female participants, men showed higher waist/height ratio and cytokine concentrations (Supplementary Materials Table S4), and were therefore probably more prone to improvements regarding inflammation.

### 4.5. Strength and Limitations

One strength of this trial is the use of modern assessment techniques such as mechanography, which allowed a more greatly differentiated evaluation of muscle function. Moreover, since dietary records are prone to different record errors, we measured omega-3 plasma index to control for participants’ adherence to omega-3 supplementation.

Limitations of our study may comprise the small sample size and the relatively short intervention time. However, this was a first pilot trial following an explorative approach to determine combined effects of a triple intervention in a three-arm study design on muscle power, inflammation and muscle biomarkers in community-dwelling old adults. Another limitation of our study may be the extent of vibration exercises (one session weekly) to improve muscle power.

## 5. Conclusions

Eight weeks of vibration and resistance exercise combined with a high-protein, omega-3-enriched diet is associated with increased muscle power and reduced inflammation in community-dwelling old men. IGF-1 increased in the omega-3-supplemented group only. Overall, a high-protein diet combined with exercise improved leg strength and CRT time. Significant sex-specific differences occurred regarding improvements in muscle function and inflammatory response, which should be further investigated in larger future trials.

## Figures and Tables

**Table 1:** Subject characteristics, body composition, physical function, and biomarkers compared between before (V1) and after (V2) eight weeks of control, high-protein (protein) and high-protein plus omega-3 (omega-3) diet.

	Control (*n* = 20)		*p*-Value ^¥^	Protein (*n* = 20)		*p*-Value ^¥^	Omega-3 (*n* = 21)		*p*-Value ^¥^	*p*-Value ^†^
	V1	V2	(V1 vs. V2)	V1	V2	(V1 vs. V2)	V1	V2	(V1 vs. V2)	(V1)
Sex (women/men)	10/10			11/9			11/10			0.895
Age (years)	69.9 ± 4.5			71.5 ± 4.6			70.4 ± 5.1			0.561
Waist/height ratio	0.58 ± 0.06	0.57 ± 0.06	0.287	0.60 ± 0.05	0.60 ± 0.06	0.110	0.59 ± 0.06	0.59 ± 0.05	0.653	0.355
BMI (kg/m^2^)	26.9 ± 2.7	26.9 ± 2.7	0.918	28.2 ± 2.3	28.4 ± 2.4	0.284	27.8 ± 2.7	28.0 ± 2.7	0.011	0.249
FFMI (kg/m^2^)	18.1 ± 2.1	18.0 ± 2.2	0.101	18.8 ± 2.1	19.2 ± 2.2	0.144	18.4 ± 2.4	18.8 ± 2.5	0.146	0.628
Grip strength (kg/BMI)	1.21 (0.59)	1.20 (0.59)	0.627	1.00 (0.43)	0.95 (0.46)	0.260	1.15 (0.57)	1.13 (0.56)	0.794	0.272
Gait speed (m/s)	1.33 (0.25)	1.30 (0.25)	0.204	1.37 (0.29)	1.29 (0.30)	0.351	1.31 (0.22)	1.32 (0.22)	0.024	0.817
Muscle power (watt/m^2^)	308 ± 68	311 ± 66	0.625	280 ± 68	289 ± 74	0.292	297 ± 62	317 ± 69	0.001	0.929
CRT time (s)	4.68 (1.55)	5.46 (1.60)	0.184	5.13 (1.29)	4.39 (1.27)	0.057	4.60 (1.13)	4.51 (0.76)	0.823	0.300
Leg strength (kg/m^2^) *	22.2 ± 6.6	22.5 ± 6.3	0.716	20.6 ± 6.6	23.9 ± 7.4	0.004	22.4 ± 4.0	24.4 ± 6.0	0.029	0.566
Omega-3 plasma index (%)	5.1 ± 1.2	4.5 ± 1.2	0.015	5.6 ± 1.7	4.7 ± 1.1	0.006	4.9 ± 1.4	8.6 ± 1.9	<0.001	0.286
CRP (mg/mL)	1.20 (1.63)	1.00 (1.57)	0.765	1.23 (2.10)	1.07 (1.90)	0.268	2.21 (3.12)	1.52 (2.82)	0.478	0.464
IL-6 (pg/mL)	2.97 (1.34)	2.98 (1.32)	0.911	2.62 (1.65)	2.62 (1.08)	0.911	3.01 (2.10)	2.56 (1.72)	0.004	0.625
IL-10 (pg/mL)	8.21 (4.28)	8.58 (3.82)	0.627	7.84 (3.55)	8.12 (3.90)	0.687	9.43 (4.21)	8.02 (4.22)	0.001	0.311
IL-6/IL-10 ratio	0.37 (0.38)	0.35 (0.33)	0.911	0.34 (0.25)	0.28 (0.18)	0.575	0.34 (0.23)	0.31 (0.20)	0.821	0.979
HMGB-1 (ng/mL)	0.38 (1.29)	0.23 (0.80)	0.286	0.29 (0.50)	0.26 (0.36)	0.723	0.25 (0.64)	0.20 (0.26)	0.006	0.797
IGF-1 (ng/mL)	204.7 ± 55.5	206.1 ± 48.8	0.814	205.5 ± 62.1	215.1 ± 70.9	0.492	230.4 ± 56.6	259.2 ± 63.7	0.004	0.277
IGFBP-3 (mg/mL)	4.35 (1.37)	4.43 (1.86)	0.478	3.91 (2.55)	3.56 (4.44)	0.370	4.92 (2.83)	5.32 (3.36)	0.085	0.272
IGF-1/IGFBP-3 ratio	47.0 ± 15.1	47.6 ± 16.1	0.840	55.3 ± 20.8	54.0 ± 21.4	0.741	48.8 ± 13.7	49.8 ± 15.4	0.744	0.272
Myostatin (ng/mL)	2.52 (0.93)	2.85 (1.40)	0.351	2.36 (1.60)	2.50 (1.38)	0.709	2.72 (1.08)	2.90 (1.52)	0.821	0.981

Values presented as mean ± standard deviation or median (interquartile range). * One male subject excluded due to measurement error. ^†^ Between-group comparison at baseline (V1) with analysis of variance or Kruskal-Wallis test. ^¥^ Within-group comparison before and after treatment (V1 vs. V2) with paired *t*-test or Wilcoxon rank test. BMI—body mass index; CRP—c-reactive protein; CRT—chair rise test; FFMI—fat-free mass index; HMGB-1—high-mobility group box-1; IGF-1—insulin-like growth factor-1; IGFBP-3—IGF-binding protein-3; IL—interleukin.

**Table 2:** Baseline values and effects of eight weeks of high-protein (protein) or high-protein plus omega-3 (omega-3) diet in comparison to control on muscle parameters, body composition and biomarkers.

	Baseline Values				Mixed Models with Interaction Effects ^#^					
	Control (*n* = 20)	Protein (*n* = 20)	Omega-3 (*n* = 21)		Protein Effects (Protein vs. Control)		Combined Effects (Omega-3 vs. Control)		Omega-3 Additional Effects (Omega-3 vs. Protein)	
Outcome Variable				*p*-Value ^†^	β (SE)	*p*-Value	β (SE)	*p*-Value	β (SE)	*p*-Value
Muscle power (watt/m^2^)	308 ± 68	280 ± 68	297 ± 62	0.929	4.688 (11.632)	0.688	16.469 (10.452)	0.118	12.572 (9.381)	0.184
CRT time (s)	4.68 (1.55)	5.13 (1.29)	4.60 (1.13)	0.300	−0.939 (0.355)	0.009	−0.420 (0.352)	0.236	0.431 (0.303)	0.159
Leg strength (kg/m^2^)	22.2 ± 6.6	20.6 ± 6.6	22.4 ± 4.0	0.566	3.109 (1.388)	0.027	1.965 (1.266)	0.124	−1.177 (1.503)	0.436
FFMI (kg/m^2^)	18.1 ± 2.1	18.8 ± 2.1	18.4 ± 2.4	0.628	0.517 (0.252)	0.042	0.581 (0.236)	0.015	0.043 (0.279)	0.877
IL-6 (pg/mL)	2.97 (1.34)	2.62 (1.65)	3.01 (2.10)	0.625	−0.030 (0.044)	0.500	−0.078 (0.040)	0.056	−0.048 (0.034)	0.161
IL-10 (pg/mL)	8.21 (4.28)	7.84 (3.55)	9.43 (4.21)	0.311	−0.017 (0.030)	0.563	−0.046 (0.023)	0.050	−0.028 (0.025)	0.252
IL-6/IL-10 ratio	0.37 (0.38)	0.34 (0.25)	0.34 (0.23)	0.979	−0.013 (0.047)	0.792	−0.032 (0.043)	0.464	−0.020 (0.035)	0.579
HMGB-1 (ng/mL)	0.38 (1.29)	0.29 (0.50)	0.25 (0.64)	0.797	0.076 (0.158)	0.631	−0.152 (0.143)	0.287	−0.227 (0.133)	0.093
IGF-1 (ng/mL)	204.7 ± 55.5	205.5 ± 62.1	230.4 ± 56.6	0.277	0.006 (0.031)	0.840	0.044 (0.020)	0.029	0.037 (0.032)	0.241
IGFBP-3 (mg/mL)	4.35 (1.37)	3.91 (2.55)	4.92 (2.83)	0.272	0.025 (0.038)	0.518	0.046 (0.035)	0.183	0.022 (0.037)	0.584
IGF-1/IGFBP-3 ratio	47.0 ± 15.1	55.3 ± 20.8	48.8 ± 13.7	0.272	−0.018 (0.044)	0.687	−0.003 (0.039)	0.945	0.015 (0.041)	0.716
Myostatin (ng/mL)	2.52 (0.93)	2.36 (1.60)	2.72 (1.08)	0.981	−0.030 (0.035)	0.390	−0.011 (0.035)	0.757	0.019 (0.031)	0.538

Values at baseline presented as mean ± standard deviation or median (interquartile range). ^†^ Between-group comparison at baseline with analysis of variance or Kruskal-Wallis test. **^#^** *p*-values for the comparison among the groups from baseline to 8 weeks obtained from generalized linear mixed models with random effects on subjects, adjusted for age, sex, and physical activity. Please note: Biomarkers are shown as absolute values at baseline, but have been log-transformed before mixed model analyses. Data presented as beta-coefficient (β) with standard error (SE). CRT—chair rise test; FFMI—fat-free mass index; HMGB-1—high-mobility group box-1; IGF-1—insulin-like growth factor-1; IGFBP-3—IGF-binding protein-3; IL—interleukin.

## Data Availability

The data that support the findings of this study are available from the corresponding author upon reasonable request.

## Supplementary Materials

The following supporting information can be downloaded at: https://www.mdpi.com/article/10.3390/nu14204274/s1, Figure S1: Flow chart of recruitment; Table S1: Daily dietary intake, physical activity, home-based exercises, and vibration frequency compared between before (V1) and after (V2) eight weeks of control, high-protein (protein) and high-protein plus omega-3 (omega-3) diet; Table S2: Baseline values and effects of eight weeks of high-protein (protein) or high-protein plus omega-3 (omega-3) diet in comparison to control on muscle parameters, body composition and biomarkers displayed for female participants; Table S3: Baseline values and effects of eight weeks of high-protein (protein) or high-protein plus omega-3 (omega-3) diet in comparison to control on muscle parameters, body composition and biomarkers displayed for male participants; Table S4: Key characteristics compared between female and male participants.
