# Supplementary material for: Effects of an Omega-3 Supplemented, High-Protein Diet in Combination with Vibration and Resistance Exercise on Muscle Power and Inflammation in Old Adults: A Pilot Randomized Controlled Trial
Source: Nutrients. 2022 Oct 13;14(20):4274. doi: 10.3390/nu14204274 (PMC9609960; doi:10.3390/nu14204274)
Supplement: Supplementary file 1 [file nutrients-14-04274-s001.zip › nutrients-1945885-supplementary.pdf]

Table S1. Daily dietary intake, physical activity, home-based exercises, and vibration frequency compared between before (V1) and after (V2) eight weeks of control, high-protein (protein) and high-protein plus omega-3 (omega-3) diet.

	**Control (*n* = 20)**		**Protein (*n* = 20)**		**Omega-3 (*n* = 21)**		***p*-value †**	***p*-value §**
	**V1**	**V2**	**V1**	**V2**	**V1**	**V2**	**(V1)**	**(Group × time)**
Energy (kcal)	1899 ± 339	1986 ± 450	1966 ± 523	2195 ± 433	1958 ± 494	2028 ± 412	0.881	0.336
Carbohydrates (En%)	37.9 ± 6.1	35.8 ± 5.2	40.1 ± 6.1	34.9 ± 4.8	41.6 ± 7.8	35.9 ± 4.8	0.215	0.144
Protein (g/kg bw)	0.96 ± 0.19	0.97 ± 0.26	0.97 ± 0.20	1.73 ± 0.27 [a]	0.93 ± 0.21	1.56 ± 0.36 [a]	0.809	<0.001
Leucine (g)	5.6 ± 1.3	5.8 ± 1.7	5.8 ± 2.3	11.9 ± 1.7 [a]	5.6 ± 1.4	10.8 ± 2.3 [a]	0.851	<0.001
Fat (En%)	43.3 ± 5.3	45.8 ± 4.7	40.2 ± 5.8	37.3 ± 5.5 [a]	39.5 ± 7.2	37.2 ± 5.1 [a]	0.127	0.036
Omega-6 (g)	9.8 (7.3)	12.7 (8.9)	10.3 (4.9)	10.7 (8.0)	8.5 (5.5)	11.2 (5.5)	0.482	0.886
Omega-3 (g)	3.1 (2.1)	3.2 (3.4)	2.4 (3.7)	3.0 (3.2)	2.7 (2.1)	5.0 (2.8)	0.371	0.008
EPA (mg)	185 (503)	195 (395)	65 (165)	80 (258)	80 (250)	830 (370) [a,b]	0.512	<0.001
DHA (mg)	235 (565)	245 (453)	145 (260)	215 (375)	150 (260)	1570 (385) [a,b]	0.309	<0.001
Sedentary activity (min)	7539 ± 634	6966 ± 1077	7477 ± 674	7195 ± 910	7633 ± 730	7477 ± 755	0.764	0.170
Light activity (min)	2123 ± 677	1777 ± 614	2266 ± 578	2098 ± 665	2196 ± 621	1958 ± 531	0.772	0.246
Moderate activity (min)	197 ± 126	168 ± 133	171 ± 139	155 ± 132	165 ± 127	172 ± 117	0.720	0.461
Vigorous activity (min)	0 (0)	0 (0)	0 (1)	0 (1)	0 (2)	0 (4)	0.211	0.914
Cross crunches (n)	30 (26)	44 (27)	30 (38)	44 (39)	40 (70)	54 (65)	0.433	0.394
Marching (n)	40 (20)	52 (20)	50 (43)	61 (40)	40 (70)	54 (60)	0.694	0.303
Squats (n)	18 (9)	31 (8)	18 (10)	29 (10)	20 (15)	34 (17)	0.636	0.893
Chair rises (n)	15 (11)	29 (10)	20 (16)	34 (23)	20 (13)	34 (15)	0.190	0.639
Chair dips (n)	16 (10)	24 (8)	15 (10)	29 (9)	20 (10)	30 (16)	0.203	0.080
Vibration frequency (Hz)	20.0 (4.5)	33 (0)	20.5 (3.4)	33 (0)	20.0 (5.5)	33 (0)	0.922	0.828

Values presented as mean ± standard deviation or median (interquartile range). † Group comparison at baseline (V1) with analysis of variance (ANOVA) or Kruskal-Wallis test. § Group comparison over time with repeated measures ANOVA. [a] Significantly different to control group. [b] Significantly different to protein group. bw body weight; DHA docosahexaenoic acid; En% energy percent; EPA eicosapentaenoic acid.

Table S2. Baseline values and effects of eight weeks of high-protein (protein) or high-protein plus omega-3 (omega-3) diet in comparison to control on muscle parameters, body composition and biomarkers displayed for female participants.

WOMEN	Baseline values				Mixed-models with interaction effects #					
	Control (*n* = 10)	Protein (*n* = 11)	Omega-3 (*n* = 11)		Protein effects (Protein vs Control)		Combined effects (Omega-3 vs Control)		Omega-3 additional effects (Omega-3 vs Protein)	
**Outcome variable**				***p*-value †**	**β (SE)**	***p*-value**	**β (SE)**	***p*-value**	**β (SE)**	***p*-value**
Muscle power (watt/m²)	232 (45)	264 (100)	252 (84)	0.629	−6.272 (15.355)	0.685	3.661 (15.499)	0.814	5.051 (11.307)	0.657
CRT-time (s)	5.52 (2.33)	4.74 (1.34)	4.84 (0.65)	0.410	−0.395 (0.441)	0.374	−0.390 (0.496)	0.435	0.190 (0.364)	0.604
Leg strength (kg/m²)	18.4 (6.5)	20.8 (8.1)	20.1 (7.2)	0.346	1.271 (1.612)	0.434	1.592 (1.292)	0.223	−1.141 (1.391)	0.416
FFMI (kg/m²)	16.0 (1.2)	17.6 (2.2)	16.5 (1.5)	0.164	0.570 (0.272)	0.041	0.631 (0.326)	0.058	0.034 (0.371)	0.927
IL-6 (pg/mL)	2.97 (1.55)	2.09 (1.28)	2.86 (2.07)	0.212	0.043 (0.053)	0.426	0.015 (0.049)	0.759	−0.014 (0.038)	0.710
IL-10 (pg/mL)	7.73 (5.59)	7.86 (3.44)	7.66 (4.24)	0.855	−0.048 (0.038)	0.207	−0.058 (0.022)	0.010	−0.007 (0.026)	0.776
IL-6/IL-10 ratio	0.46 ± 0.30	0.29 ± 0.13	0.36 ± 0.16	0.165	0.093 (0.055)	0.093	0.069 (0.048)	0.158	−0.007 (0.038)	0.851
HMGB-1 (ng/mL)	0.50 (1.13)	0.20 (0.55)	0.13 (0.53)	0.170	0.276 (0.230)	0.236	0.079 (0.169)	0.642	−0.152 (0.129)	0.242
IGF-1 (ng/mL)	175.9 (37.0)	201.7 (67.9)	219.2 (89.6)	0.096	0.011 (0.035)	0.751	0.049 (0.031)	0.112	0.046 (0.036)	0.211
IGFBP-3 (mg/mL)	3.89 (1.97)	3.89 (2.26)	3.70 (3.08)	0.757	0.044 (0.052)	0.405	0.079 (0.043)	0.069	0.054 (0.040)	0.178
IGF-1/IGFBP-3 ratio	48.1 ± 16.1	62.5 ± 19.1	55.0 ± 12.5	0.143	-0.033 (0.044)	0.466	−0.028 (0.049)	0.569	−0.008 (0.045)	0.854
Myostatin (ng/mL)	2.74 (3.10)	2.12 (1.01)	2.72 (1.18)	0.502	0.011 (0.042)	0.797	0.044 (0.043)	0.307	0.041 (0.031)	0.194

Values at baseline presented as mean ± standard deviation or median (interquartile range). † Between-group comparison at baseline with analysis of variance (ANOVA) or Kruskal-Wallis test. # *p*-values for the comparison among the groups from baseline to 8 weeks obtained from generalized linear mixed models with random effects on subjects, adjusted for age, sex, and physical activity. Please note: Biomarkers are shown as absolute values at baseline, but have been log-transformed before mixed-model analysis. Data presented as beta-coefficient (β) with (standard error (SE). CRT chair rise test; FFMI fat-free mass index; HMGB-1 high-mobility group box-1; IGF-1 insulin-like growth factor-1; IGFBP-3 IGF-binding protein-3; IL interleukin.

Table S3. Baseline values and effects of eight weeks of high-protein (protein) or high-protein plus omega-3 (omega-3) diet in comparison to control on muscle parameters, body composition and biomarkers displayed for male participants.

M E N	Baseline values				Mixed-models with interaction effects #					
	Control (n = 10)	Protein (n = 9)	Omega-3 (n = 10)		Protein effects (Protein vs Control)		Combined effects (Omega-3 vs Control)		Omega-3 additional effects (Omega-3 vs Protein)	
Outcome variable				*p*-value †	β (SE)	*p*-value	β (SE)	*p*-value	β (SE)	*p*-value
Muscle power (watt/m²)	361 ± 34	297 ± 81 [a]	341 ± 37	0.045	17.065 (17.223)	0.327	28.376 (13.576)	0.042	21.606 (10.088)	0.037
CRT-time (s)	4.54 ± 0.73	6.17 ± 1.95 [a,b]	4.37 ± 0.83	0.009	−1.532 (0.543)	0.007	−0.388 (0.481)	0.424	0.689 (0.372)	0.069
Leg strength (kg/m²)	25.4 ± 5.3	19.5 ± 7.5	24.1 ± 3.1	0.078	4.417 (1.839)	0.020	3.064 (2.085)	0.148	−1.714 (2.085)	0.415
FFMI (kg/m²)	19.9 ± 1.3	20.6 ± 1.2	20.6 ± 1.1	0.314	0.533 (0.443)	0.235	0.501 (0.312)	0.115	0.139 (0.281)	0.623
IL-6 (pg/mL)	2.97 (1.40)	3.69 (2.52)	3.33 (4.02)	0.722	−0.100 (0.064)	0.124	−0.176 (0.057)	0.003	−0.085 (0.038)	0.030
IL-10 (pg/mL)	8.74 (6.79)	7.81 (5.17)	10.27 (7.24)	0.182	0.024 (0.039)	0.550	−0.047 (0.039)	0.237	−0.052 (0.026)	0.053
IL-6/IL-10-ratio	0.40 ± 0.32	0.47 ± 0.20	0.37 ± 0.16	0.658	−0.125 (0.067)	0.066	−0.129 (0.062)	0.042	−0.033 (0.044)	0.457
HMGB-1 (ng/mL)	0.22 (1.37)	0.34 (0.61)	0.36 (0.83)	0.586	−0.088 (0.162)	0.588	−0.434 (0.212)	0.046	−0.315 (0.179)	0.084
IGF-1 (ng/mL)	238.8 ± 54.7	201.4 ± 67.6	242.7 ± 64.6	0.304	0.004 (0.054)	0.939	0.034 (0.025)	0.184	0.028 (0.033)	0.409
IGFBP-3 (mg/mL)	4.71 (3.79)	4.41 (5.38)	5.69 (2.50)	0.366	0.000 (0.053)	0.997	0.012 (0.053)	0.825	−0.014 (0.046)	0.757
IGF-1/IGFBP-3-ratio	46.0 ± 14.8	46.4 ± 20.3	42.0 ± 12.2	0.798	0.002 (0.078)	0.981	0.024 (0.060)	0.689	0.042 (0.051)	0.413
Myostatin (ng/mL)	2.44 ± 0.61	3.62 ± 1.83	2.96 ± 1.42	0.189	−0.078 (0.053)	0.150	−0.062 (0.053)	0.248	−0.039 (30.857)	>0.999

Values at baseline presented as mean ± standard deviation or median (interquartile range). † Between-group comparison at baseline with analysis of variance (ANOVA) or Kruskal-Wallis test. [a] Significantly different to control group. [b] Significantly different to omega-3 group. # p-values for the comparison among the groups from baseline to 8 weeks obtained from generalized linear mixed models with random effects on subjects, adjusted for age, sex, and physical activity. Please note: Biomarkers are shown as absolute values at baseline, but have been log-transformed before mixed-model analysis. Data presented as beta-coefficient (β) with (standard error). CRT chair rise test; FFMI fat free mass index; HMGB-1 high-mobility group box-1; IGF-1 insulin-like growth factor-1; IGFBP-3 IGF-binding protein-3; IL interleukin.

Table S4. Key characteristics compared between female and male participants.

	**Women (n = 32)**	**Men (n = 29)**	***p*-value**
Waist/height ratio	0.58 ± 0.05	0.61 ± 0.06	0.064
Muscle power (watt/m^2)	252.2 (79.7)	343.0 (58.6)	<0.001
Leg strength (kg/m^2) *	19.6 (8.9)	24.2 (5.1)	0.022
IL-6 (pg/mL)	2.39 (1.07)	3.19 (1.93)	0.011
IL-10 (pg/mL)	7.75 (3.14)	9.43 (4.89)	0.038
IL-6/IL-10 ratio	0.37±0.21	0.41±0.23	0.474

Values presented as mean ± standard deviation or median (interquartile range) and compared using *t*-Test or Mann-Whitney U-Test. * One male subject excluded due to measurement error. IL interleukin.
